# Copula miss-specification in REML multivariate genetic animal model estimation

**DOI:** 10.1186/s12711-022-00729-3

**Published:** 2022-05-26

**Authors:** Tom Rohmer, Anne Ricard, Ingrid David

**Affiliations:** 1 GenPhySE, Université de Toulouse, INRAE, ENVT, 31326 Castanet Tolosan, France; 2 Université Paris-Saclay, INRAE, AgroParisTech, GABI, Jouy-en-Josas, France; 3 Institut Français du Cheval et de l’Equitation, Pôle Développement, Innovation et Recherche, Exmes, France

## Abstract

**Background:**

In animal genetics, linear mixed models are used to deal with genetic and environmental effects. The variance and covariance terms of these models are usually estimated by restricted maximum likelihood (REML), which provides unbiased estimators. A strong hypothesis of REML estimation is the multi-normality of the response variables. However, in practice, even if the marginal distributions of each phenotype are normal, the multi-normality assumption may be violated by non-normality of the cross-sectional dependence structure, that is to say when the copula of the multivariate distribution is not Gaussian. This study uses simulations to evaluate the impact of copula miss-specification in a bivariate animal model on REML estimations of variance components.

**Result:**

Bivariate phenotypes were simulated for populations undergoing selection, considering different copulas for the dependence structure between the error components. Two multi-trait situations were considered: two phenotypes were measured on the selection candidates, or only one phenotype was measured on the selection candidates. Three generations with random selection and five generations with truncation selection based on estimated breeding values were simulated. When selection was performed at random, no significant differences were observed between the REML estimations of variance components and the true parameters even for the non-Gaussian distributions. For the truncation selections, when two phenotypes were measured on candidates, biases were systematically observed in the variance components for high residual dependence in the case of non-Gaussian distributions, especially in the case of a heavy-tailed or asymmetric distribution when the two traits were measured. Conversely, when only one phenotype was measured on candidates, no difference was observed between the Gaussian and non-Gaussian distributions in REML estimations.

**Conclusions:**

This study confirms that REML can be used by geneticists to evaluate breeding values in the multivariate case even if the multivariate phenotypes deviate from normality in the situation of random selection or if one trait is not measured for the candidate under selection. Nevertheless, when the two traits are measured, the violation of the normality assumption may lead to non-negligible biases in the REML estimations of the variance-covariance components.

**Supplementary Information:**

The online version contains supplementary material available at 10.1186/s12711-022-00729-3.

## Background

Multivariate mixed models are widely used in animal genetics to deal with genetic and environmental effects (see for example Mrode [1], Jiang [2], Meyer [3]). Even though many methods are available to estimate the variance and covariance terms of these models (see Jensen and Mao [4] for an exhaustive review), in practice, these parameters are frequently estimated using restricted maximum likelihood (REML), which a method that was developed by Patterson and Thompson [5] and Verbyla [6] and provides unbiased estimators. Best linear unbiased predictions (BLUP) are then used to estimate the breeding values and to perform selection [7].

A strong assumption of REML estimations is the multi-normality of the vector of phenotypes. However, this assumption may be violated even if the marginal distributions are normal. Indeed, marginal distributions are not sufficient to characterize the multivariate distribution of a random vector. The copula of a random vector is a (multivariate) cumulative distribution function that links the marginal distributions of the random vector to the multivariate distribution, and characterizes the dependence structure of the random vector. In recent years, copulas have been extensively used in many fields including hydrology [8], actuarial sciences and finance [9]. In genetic studies, Tregouet et al. [10] used copula (Frank’s family) to analyze familial binary data and Li et al. [11] used normal copulas to analyze non-Gaussian bivariate traits, in a quantitative trait linkage context. An exhaustive study of copula can be found in Nelson [12]. To our knowledge, in genetics, even when the distribution of the bivariate phenotypes is not normal, REML estimations of the variance components are still used in spite of the fact that the theoretical consistency of the estimator is not established.

The aim of the present study was to use simulations to evaluate the impact of a copula miss-specification in the bivariate animal model on REML estimations of variance-covariance components and on estimated breeding values (EBV) when each of the two traits of the bivariate model are Gaussian but the set of bivariate traits is not.

## Method

As mentioned above, marginal distributions do not enable the characterization of the multivariate distribution of a random vector. Let $\varvec{\mathrm {Z}} = (Z_1,Z_2)$ be a 2-dimensional random vector with cumulative distribution function (cdf) *F* and let $F_1,F_2$ be the marginal cdf of $\varvec{\mathrm {Z}}$ assumed to be continuous. According to Sklar’s theorem [13], there is a unique function $C:[0,1]^d\rightarrow [0,1]$ such that:

1 $$\begin{aligned} F(\varvec{\mathrm z}) = C\{F_1(z_1),F_2(z_2)\}, \qquad \varvec{\mathrm {z}}=(z_1,z_2)\in {\mathbb {R}}^2. \end{aligned}$$

The copula *C* characterizes the dependence structure of vector $\varvec{\mathrm {Z}}$. For $Z_1$ and $Z_2$ Gaussian variables, the vector $(Z_1,Z_2)$ is a Gaussian vector if the copula of $(Z_1,Z_2)$ is the normal (N) copula given by:

$$\begin{aligned} C_\rho ^N(u,v) = \Phi _\rho (\Phi ^{-1}(u),\Phi ^{-1}(v)),\quad (u,v)\in [0,1]^2, \end{aligned}$$

where $\Phi _\rho$ stands for the cdf of the centered bivariate normal distribution with a correlation matrix whose off-diagonal entries are $\rho \in (-1,1)$ and $\Phi ^{-1}$ the inverse cdf of the standard normal distribution. Other standard bivariate copulas are the Frank (F) copula, the Clayton (Cl) copula and the Joe (J) copula, given for $(u,v)\in [0,1]^2$ respectively, by:

$$\begin{aligned} C_{\theta }^F(u,v)&= \frac{1}{\theta } \log \left( 1+\frac{(\exp (-u \theta )-1)(\exp (-v \theta )-1)}{\exp (-\theta -1)}\right) ,\quad \theta \in {\mathbb {R}}^\star ,\\ C_{\theta }^{Cl}(u,v)&= \max \left( \left( u^{-\theta }+v^{-\theta }-1\right) ^{-1 / \theta }, 0\right) ,\quad \theta \in [-1,0)\cup (0,+\infty ),\\ C_{\theta }^{J}(u, v)&=1-\left[ (1-u)^{\theta }+(1-v)^{\theta }-(1-u)^{\theta }(1-v)^{\theta }\right] ^{1 / \theta }\quad \theta \ge 1. \end{aligned}$$

A more exhaustive list of copulas can be found in the books of Nelson [12] and Joe [14]. The well-known Kendall’s correlation can be defined in terms of the copula $C_\theta$ by:

$$\begin{aligned} \tau = 4\int _{[0,1]^2}C_{\theta }(u,v) dC_{\theta }(u,v) - 1, \end{aligned}$$

see Genest and Favre [15] for an exhaustive discussion of the relation between dependence measure and copulas. Because $C_\theta ^{Cl}$ and $C_\theta ^J$ are not defined for a negative Kendall’s correlation, the rotated 270$^\circ$ copulas were used to deal with the negative correlations. The formal expression of the rotated copulas is:

$$\begin{aligned} {}^r \!C_\theta \left( u_{1}, u_{2}\right) =u_{1}-C_\theta \left( u_{1}, 1-u_{2}\right) . \end{aligned}$$

**Fig. 1:**
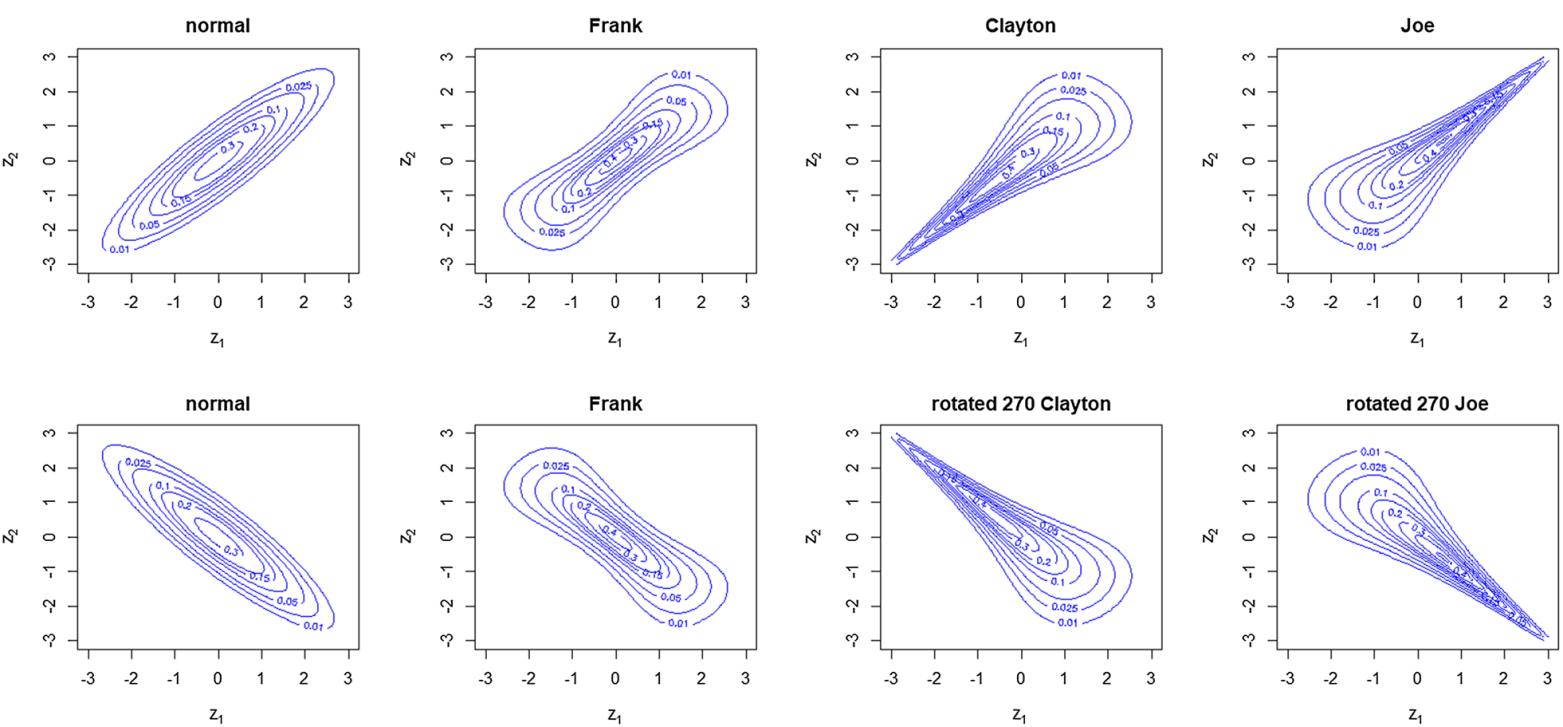
Contour plot of bivariate distributions with Gaussian margins and for copula, the normal copula, Frank’s copula, Clayton’s copula and Joe’s copula or the rotated versions, with Kendall’s tau (top) $\tau =0.7$ (bottom) $\tau =-0.7$

As an illustration, Fig. 1 shows the contour plot of bivariate distributions with Gaussian margins and N, F, C, J copula (or rotated version) with Kendall’s tau of $\pm 0.7$.

N and F copulas are symmetric, whereas both Cl and J copulas are asymmetric (in the sense of radial asymmetry [16]).

In a univariate setting, skewness and kurtosis measure the asymmetry of the distribution and the heaviness of the distribution tails, respectively, and make it possible to quantify the distance from normality. An extension of these measures for the multivariate case is proposed in Mardia [17]. For the bivariate Gaussian case, Mardia’s skewness and kurtosis are 0 and 8, respectively, regardless of the variance-covariance parameters. Mardia’s skewness and kurtosis were evaluated using 1000 Monte Carlo simulations for the bivariate distributions presented in Fig. 1 and for a sample of size $n=1000$. Their values are listed in Table 1. As expected, these bivariate distributions are more distant from a bivariate Gaussian distribution when Kendall’s tau is high. The bivariate distribution with J copula appeared to have higher skewness and kurtosis values than the other copulas.

**Table 1:** Mean value of Mardia’s skewness and kurtosis for the simulated bivariate distributions

	$\tau =0.4$		$\tau =0.7$	
	Skewness	Kurtosis	Skewness	Kurtosis
N	0.02	7.98	0.02	7.99
F	0.03	8.66	0.07	11.01
Cl	0.69	8.61	2.31	11.58
J	0.78	8.73	2.35	11.67

Mean values were obtained from 1000 simulations of a sample of size 1000. Marginal distributions were Gaussian. Copulas were normal (N), Frank (F), Clayton (Cl) and Joe (J)

Other standard measures in a multivariate setting are lower ($\lambda _L$) and upper ($\lambda _U$) tail dependence. These indexes between 0 and 1 measure the correlations in the (bottom) left and (top) right tail of the distribution and are more often used in an extreme value context. Concepts of lower and upper tail dependence can be found in Joe [14]. The Cl copula has a lower tail dependence but no upper tail dependence (for $\tau =0.7$, their values are $\lambda _L=0.86$ and $\lambda _U=0$). The J copula has an upper tail dependence but no lower tail dependence (for $\tau =0.7$, their values are $\lambda _U=0$ and $\lambda _L=0.86$). Neither F nor N have a tail dependence (regardless of Kendall’s tau, $\lambda _U=0$ and $\lambda _L=0$).

### Illustrations

The normality of the dependence structure between traits (precorrected by environmental factors), can be graphically evaluated by fitting a bivariate normal contour plot to the plot of the two phenotypes (or, if the phenotypes are not marginally distributed, the scatter plot of Gaussian quantiles of the rank of the two phenotypes over the number of observations, see for example [15]). Deviations from normality can also be evaluated using Mardia’s skewness and kurtosis as mentioned before. Such a graphic and measures are illustrated in the two following examples.

#### Example 1

The first illustrative dataset consists in $n=2808$ Large White pigs, observed over a 100-day period between 2017 and 2019. The phenotypes observed were cumulative feed intake (CFI) at 10 days and average daily gain (ADG) at 100 days. The Pearson’s correlation between the two phenotypes is $\rho = -0.27$.

The quantiles of the ranks over *n* of the phenotypes and the contour plot of the bivariate normal distribution are plotted in Fig. 1a. In spite of the normality of the margins, the distribution of the points is not homogeneous in the ellipse of the contour plot of the bivariate Gaussian distribution, suggesting that the hypothesis of bivariate normality of pairs of phenotypes is unrealistic.

**Fig. 2:**
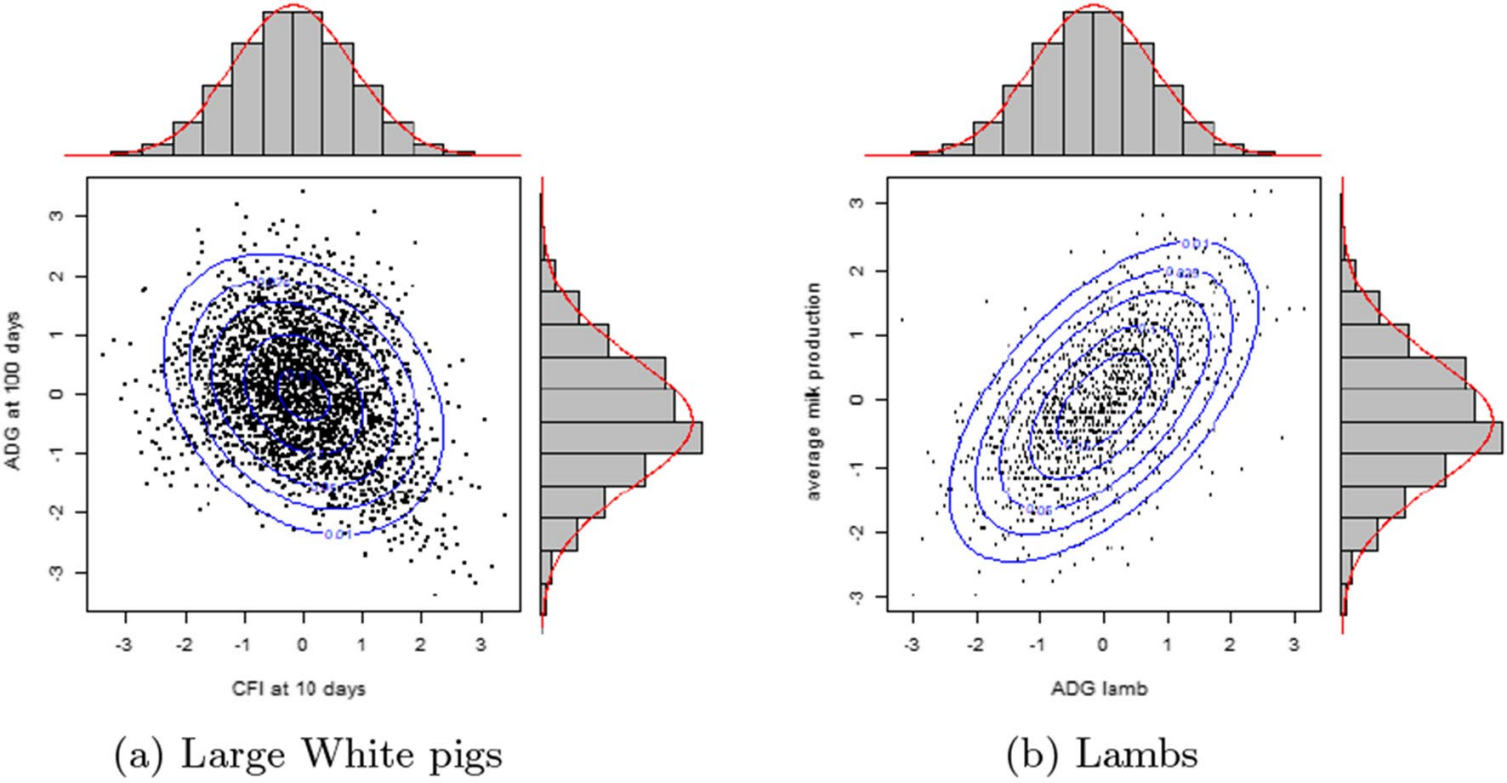
Plot of Gaussian quantiles of the ranked observations over *n* animals for the two illustrations. **a** Cumulative feed intake (CFI) at 10 days and average daily gain (ADG) at 100 days of $n=2808$ Large White pigs, **b** ADG of $n=1289$ lambs and their mothers’ average milk production. Contour plots of the normal copula with respective Pearson’s correlation $\rho$ of − 0.27 and 0.57.

#### Example 2

The second illustration is the average daily gain of $n=1289$ lambs and their mothers’ average milk production. The Pearson’s correlation between the two phenotypes is $\rho = 0.57$. The quantiles of the ranks over *n* of the phenotypes are plotted in Fig. 1b. As in Example 1, the distribution of the points is not homogeneous in the ellipse of the bivariate Gaussian distribution and the bivariate normality hypothesis of pairs of phenotypes can be questioned.

**Fig. 3:**
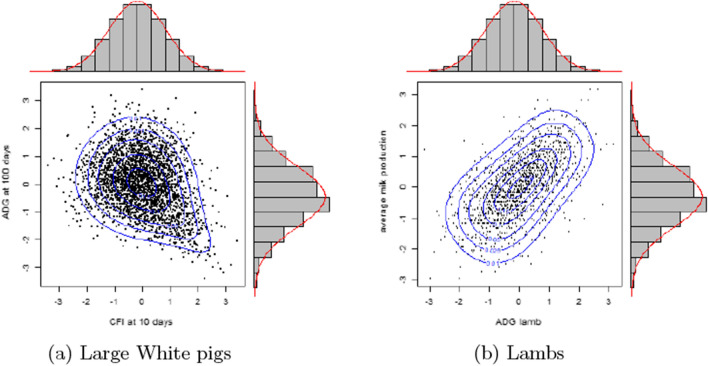
Plot of Gaussian quantiles of the ranked observations over *n* animals for the two illustrations. **a** Cumulative feed intake (CFI) at 10 days and average daily gain (ADG) at 100 days of $n=2808$ Large White pigs. Contour plots of the rotated 270$^\circ$ Joe-Frank copula with parameters $(\delta ,\lambda ) = (-1.4,-1)$. **b** ADG of $n=1289$ lambs and their mothers’ average milk production. Contours plots of the Joe-Frank copula with parameter $(\delta ,\lambda ) = (6,0.56)$

Using the VineCopula package [e.g. 18] in R [19], it is possible to define the “most adjusted” copula in terms of Akaike information criterion (AIC) and to perform the corresponding contour plots. It should be noted that, within a trait, data are considered to be independent in the calculus of the AIC, which is not true due to the genetic part. Nevertheless, this AIC classification allows us to obtain a more acceptable copula. The copula that fits both datasets best is the Joe-Frank [14] (or rotated version). In Fig. 3, the contour plots of the bivariate distribution with the Joe-Frank copula and Gaussian margins are shown for the two illustrations. The choice of these bivariate distributions seems to be more satisfactory than the multivariate Gaussian distributions shown in Fig. 2.

Mardia proposed a normality test based on the distance between the measured skewness and kurtosis and the theoretical values for the Gaussian case [17]. For the pig example, skewness and kurtosis values were, respectively, 0.40 and 8.15 and the p-values for the respective Mardia normality tests were $10^{-6}$ and 0.31. Hence, the normality hypothesis can be rejected because of the asymmetry of the distribution.

For the lamb example, the respective skewness and kurtosis values were 0.03 and 8.67 and the p-values of the respective Mardia tests were 0.14 and $2.3\times 10^{-3}$. Hence, the normality hypothesis can be rejected because of the heaviness of the distribution tails.

In this study, the impact of a non-normal dependence structure between phenotypes on the REML estimations of the variance components in the genetic models was evaluated by simulating a simplified pig breeding scheme.

### Simulations

The populations were generated following the same schema as Gonzales et al. [20]. Founders $G_0$ consisted in $n_0=216$ unrelated animals, 204 females and 12 males. For the following generations, each male was mated to 17 females. Eight generations ($G_1$ to $G_8$) were simulated. To this end, two multi-trait situations that exist in pig farming were considered.

The first situation is standard case, where all progeny had two observed phenotypes. In the simulations, each female produced 12 offspring: 2 males and 10 females (the artificial unbalanced sex ratio may be due to a higher male culling rate in unrelated other phenotypes). Thus, each generation comprised 2448 animals. All progeny were candidates for selection. The total number of animals over eight generations, including founders was $n=19,800$.

The second multi-trait situation mimicked a second phenotype that can only be measured after culling (such as carcass traits). In the simulations, half of the animals have a missing value on the second phenotype. Only the animals with a missing value are candidates for breeding. Each female produced 24 offspring: 4 males and 24 females with 2 males and 12 females with both traits and 2 males and 12 females with only one trait, the latter being the candidates for selection. Thus, each generation comprised 4896 animals, and over eight generations, $n=39,384$. Only 2448 animals per generation were candidates for selection.

In both situations, male and female breeders were randomly mated to produce the next generation, but in such a way, that the full/half siblings were not mated with each other to limit inbreeding.

In generations $G_1$ to $G_3$, the reproducers were chosen at random. Then, from $G_4$ to $G_8$, selection was performed for a balanced breeding objective for the two traits. Reproducers were chosen by truncation from a combination of their breeding values estimated by BLUP with a weight of 50% on each trait. Selection was made within the progeny of one male. Among the 34 male offspring of a male, the best male was selected, which represents a selection rate of 2.9% and among the 170 female offspring of a male, the best 17 females were selected, which represents a selection rate of 10%.

Genetic values were simulated as follows. For the founder generation, given a genetic covariance matrix $$\varvec{\Sigma } = \left( \begin{array}{ll} \sigma ^2_{a_1} &{} \sigma _{a_{12}} \\ \sigma _{a_{12}} &{} \sigma ^2_{a_2}\end{array}\right)$$, breeding vectors $(a_{i,1},a_{i,2})$, $i=1,\ldots ,n_0$ were drawn according to the Gaussian distribution with the covariance matrix $\varvec{\Sigma }$. For the other generations, the breeding values were $a_{i,j} = 0.5(a_{i_f,j} + a_{i_m,j}) + M_{ij}$, $j=1,2$ and $i=n_0+1,\ldots ,n$, where $i_f$ and $i_m$ were respectively the indexes of the sire and dam of the *i*th animal and $(M_{i1}, M_{i2})$ was the Mendelian sampling term drawn from a bivariate Gaussian distribution with the covariance matrix $\varvec{\Sigma }/2$.

Performances were then simulated according to different residuals. All the simulated residuals $(\varepsilon _{i,1},\varepsilon _{i,2})$, $i=1,\ldots ,n$, were sampled independently from a bivariate distribution with standard Gaussian margins. That is to say, in Eq. (1), $F_1=F_2=\Phi$. The copulas were the N copula (Gaussian case), F copula, Cl copula and J copula (or the rotated version according to the sign of the residual correlation).

Conditional copula approaches were used to generate the residuals for non-Gaussian distributions [12, Section 2.9]. For negative Kendall’s correlations, rotated 270$^\circ$ copula were used taking $-\varepsilon _{i,2}$ instead of $\varepsilon _{i,2}$.

To be sure that the comparisons would be meaningful, the copula parameter was chosen in such a way that Kendall’s tau, $\tau _e$, was the same for all residuals.

Note that, thus, the Pearson’s residual correlations $\rho _e$ between the different simulated copula were different. Given the bivariate distributions of the residual terms, theoretical correlations $\rho _e$ were obtained by numerical integration, except for the Gaussian case, where Kendall’s tau and Pearson’s correlation are linked by the following formula $\rho = \sin (\frac{\pi }{2}\tau )$ [14].

We considered three categorical fixed effects with three, two and two modalities for each trait and denoted $\varvec{\mathrm \upbeta }_{j}$ the vector of the fixed effect for the *j*th trait (arbitrary values in our simulations). The associated design matrices are denoted $\varvec{\mathrm {X}}_j$, $j=1,2.$

Finally, the bivariate phenotypes $(y_{i1},y_{i2})$, $i=1,\ldots ,n$ were obtained following the bivariate animal model:

2 $$\begin{aligned} \left\{ \begin{array}{l} \varvec{\mathrm y}_{1}=\varvec{\mathrm {X}}_{1} \varvec{\mathrm \upbeta }_{1}+\varvec{\mathrm a}_{1}+\varvec{\mathrm \upvarepsilon }_{1} \\ \varvec{\mathrm y}_{2}=\varvec{\mathrm {X}}_{2} \varvec{\mathrm \upbeta }_{2}+\varvec{\mathrm a}_{2}+\varvec{\mathrm \upvarepsilon }_{2}, \end{array}\right. \end{aligned}$$

where for $j=1,2$, $\varvec{\mathrm y}_{j} = (y_{1,j},\ldots y_{n,j})$, $\varvec{\upvarepsilon }_{j} = (\varepsilon _{1,j},\ldots \varepsilon _{n,j})$ and $\varvec{\mathrm a}_{j} = (a_{1,j},\ldots a_{n,j})$.

For each generation $G_k$, $k\ge 3$, the variances and covariances were estimated by REML using the data from generations $G_0$ to $G_k$. The heritabilities of the two traits (in the narrow sense [21]) were then computed as:

3 $$\begin{aligned} h^2_j = \frac{\sigma ^2_{{a}_j}}{\sigma ^2_{{a}_j} + \sigma ^2_{{e}_j}},\qquad j=1,2. \end{aligned}$$

The BLUP of the BV were obtained, generation after generation by solving Henderson’s mixed model equations [1, 7] again using the data from generations $G_0$ to $G_k$. The genetic gains for the generation $G_k$ for each of the two traits, were evaluated by the difference in the mean value of the BV of generation $G_k$ with the mean value of the BV of the founders ($G_0$) relative to the theoretical genetic standard deviation. The REML estimations and the BLUP were made using the Asreml software [22].

Residual variances $\sigma ^2_{e_1}$ and $\sigma ^2_{e_2}$ were set to 1. For the genetic effect, we considered two Kendall’s correlations: $\tau _a \in \{0.2, 0.4\}$, that correspond to Pearson’s correlation $\rho _a \in \{0.31, 0.59\}$. For the residual effect, we considered four Kendall’s correlations: $\tau _e \in \{\pm 0.4, \pm 0.7\}$. The corresponding Pearson’s residual correlations (in absolute values) for N, F, Cl and J copula, respectively, were for the medium correlation ($|\tau _e|=0.4$): 0.588, 0.544, 0.578 and 0.576 and for the high correlation ($|\tau _e|=0.7$): 0.891, 0.846, 0.852 and 0.850. For each possible pair of correlations ($\tau _a$ and $\tau _e$), the genetic variances ($\sigma ^2_{a_1}$ and $\sigma ^2_{a_2}$) considered were (0.18 and 0.18), (0.18 and 0.67) and (0.67 and 0.67) leading to low and moderate heritabilities of 0.153 and 0.401. Thus, 24 parameter sets were considered. Finally, 1000 Monte Carlo simulations per set (of such populations) were run and the mean value and the SE of the estimated heritabilities, genetic and residual correlations and genetic gains were considered. Note that the SE of the estimates corresponds to the SD of the 1000 estimations.

## Results

First, we present the results of the estimated heritabilities, genetic correlations and residual correlations in the situation with no missing phenotype. Then, we summarize the situation in which some phenotypes on the second trait are missing.

### Heritability, with no missing phenotypes

At generation $G_3$, (random selection), regardless of the set of parameters and copula, the absolute biases for heritability (average of the 1000 replicates) ranged between 0.000 and 0.001 with SE ranging from 0.017 to 0.031. No differences were significant for the t-test at level $\alpha =0.05$.

In Additional file 1: Tables S1, S2, the average biases and SE of the estimated heritabilities at generation $G_8$ are given for the situation with no missing phenotypes.

At generation $G_8$, regardless of the set studied, the absolute bias of the heritability for the N copulas was on average 0.003 with values ranging from 0.002 to 0.005. The absolute bias for the heritability of the non-normal copulas tended to be higher (in absolute value): on average, 0.016 with values ranging from − 0.052 to 0.059. The SE ranged from 0.007 to 0.019, with an average of 0.015 for a positive residual correlation and an average of 0.012 for a negative residual correlation.

The differences between the estimated heritabilities and the true heritabilities were never significant for the F copula. For the Cl and J copulas, when the residual dependence was positive, the differences were significant for the trait with a moderate heritability when the residual dependence was high and the heritability of the two traits differed, with an underestimated heritability for Cl and an overestimated heritability for J. The maximum of the absolute bias was 0.059 for the J copula and 0.052 for the Cl copula. When the residual dependence was negative, with some rare exceptions, the moderate heritabilities estimates were systematically biased, whereas the heritability estimate of the traits with a low heritability only differed significantly from its true value when the negative residual dependence was strong.

**Fig. 4:**
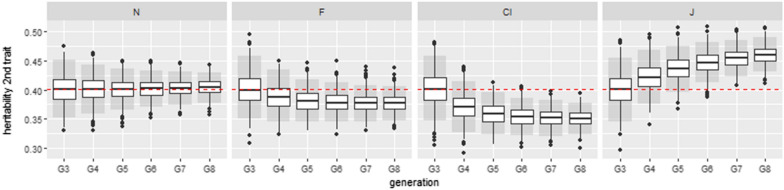
Boxplot of the estimated heritabilities from generation $G_3$ to generation $G_8$ for the set corresponding to $h_1^2=0.153$, $h_2^2=0.401$, $\tau _a= 0.4$ and $\tau _e=0.7$ with no missing phenotypes. 1000 simulations were run. The red dotted line represents the theoretical heritability. Residual copulas are normal(N), Frank (F), Clayton (Cl) and Joe (J). Grey boxes are the 95% confidence intervals

Figure 4 illustrates one of the sets of parameters with the strongest absolute biases, with a true heritability of 0.153 for the first trait and of 0.401 for the second, where the estimated heritabilities for the second trait were boxplotted generation by generation for each copula. Kendall’s tau of the genetic component was $\tau _a=0.4$ and Kendall’s tau of residual component was $\tau _e=0.7$.

For this set of parameters, we observed for the trait with the highest heritability marked changes in the estimated biases between $G_3$ and $G_4$ (the absolute difference in the mean values of the estimated biases between $G_3$ and $G_4$ was 0.031 for Cl copula). The change between $G_7$ and $G_8$ was much smaller (the absolute difference in the mean values was 0.006 for J copula). On average, Mardia’s kurtosis values obtained from the first three generations (random selection) were greater than 8.73 for all the non-normal copulas and Mardia’s skewness values were greater than 0.39 for Cl and J copula (leading to a rejection of the normal hypothesis).

### Correlations with no missing phenotypes

At generation $G_3$, for both the genetic and residual correlations, no significant differences were observed for the t-test at level $\alpha =0.05$. The absolute biases for the genetic correlations ranged from 0.000 to 0.011 (SE between 0.036 and 0.115). The absolute biaises of the residual correlations were always lower than 0.002 (SE between 0.004 and 0.026).

In Additional file 1: Tables S3, S4, the average biases and SE of the estimated correlations (genetic and residual) at generation $G_8$ are shown for the situation with no missing phenotypes.

At generation $G_8$, regardless of the cases studied, the absolute bias in the genetic correlation of the N copula was on average 0.003 and was higher for the non-normal copulas, on average 0.047. The absolute bias in the residual correlations of the N copula was on average 0.001 and for the non-normal copulas, on average 0.017. The SE ranged from 0.019 to 0.075 for the genetic correlations (lower for a positive residual correlation, on average 0.036 and higher for negative residual correlation, average 0.052) and from 0.003 to 0.015 for the residual correlations.

For non-normal copulas, when the residual dependence was positive, the genetic correlation was systematically biased upward and the residual correlation of data simulated with the F or Cl copulas was biased downward. With some rare exceptions, the reverse was observed for the data simulated using the J copula. When the residual dependence is negative, the general trend is underestimation of the genetic correlation and overestimation of the residual correlation for all non-normal copulas, but, for the genetic correlation, the values were never significantly different from 0.

In the case of a positive residual dependence, for both the residual and genetic correlations, the highest values of the absolute bias were for a high residual dependence and heritability for both traits. In these cases, the differences between estimated and true parameters for the non-Gaussian distributions were all significant. The maximum absolute bias was reached for the J copula, i.e. 0.161 for the genetic correlations and 0.06 for the residual correlations, followed by the Cl copula, 0.140 for the genetic correlations, and 0.056 for the residual correlations.

**Fig. 5:**
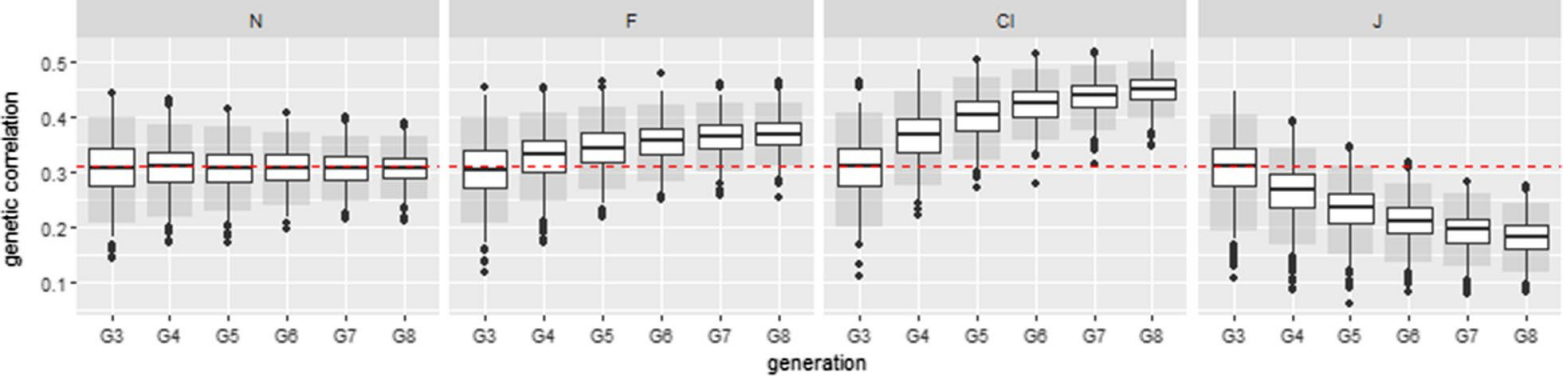
Boxplots of estimated genetic correlations from generation $G_3$ to generation $G_8$ for the set corresponding to $h_1^2=0.401$, $h_2^2=0.401$, $\tau _a= 0.2$ and $\tau _e=0.7$ with no missing phenotypes. 1000 simulations were performed. The red dotted line represents the theoretical genetic correlation. Residual copulas are normal (N), Frank (F), Clayton (Cl) and Joe (J). Grey boxes are the 95% confidence intervals

**Fig. 6:**
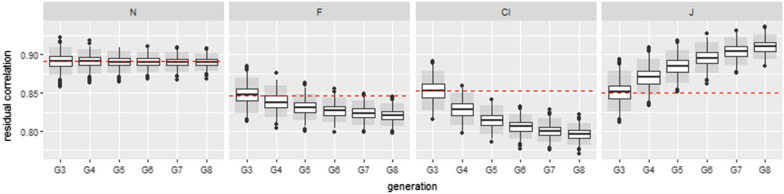
Boxplots of estimated residual correlations from generation $G_3$ to generation $G_8$ for the set corresponding to $h_1^2=0.401$, $h_2^2=0.401$, $\tau _a= 0.2$ and $\tau _e=0.7$ with no missing phenotypes. 1000 simulations were performed. The red dotted lines represent the theoretical residual correlations. Residual copulas are normal (N), Frank (F), Clayton (Cl) and Joe (J). Grey boxes are the 95% confidence intervals

Figures 5 and 6 illustrate one of the set of parameters with the strongest absolute biases for the genetic and residual correlations, with the same heritability of 0.401 for the two traits. Kendall’s tau of the genetic component was $\tau _a=0.2$ and Kendall’s tau of the residual component was $\tau _e=0.7$.

For these Kendall correlations, we observed marked differences in the estimated biases between $G_3$ and $G_4$ both for the genetic and residual correlations. The difference was much smaller between $G_7$ and $G_8$. For the genetic correlations, on average, the absolute difference in bias between $G_3$ and $G_4$ was 0.042 and 0.012 between $G_7$ and $G_8$ for the J copula. For the residual correlations, on average, the absolute difference in bias between $G_3$ and $G_4$ was 0.024 and 0.007 between $G_7$ and $G_8$, for the J copula. On average, Mardia’s kurtosis values obtained from the first three generations (random selection) were lower than 8.21 for all the non-normal copulas (the tests of multivariate normality based on kurtosis were, on average, non-significant) but Mardia’s skewness values were, on average, greater than 1.17 for the C and J copula (leading to a rejection of the normal hypothesis).

### Genetic gain with no missing phenotypes

The genetic gains for generation $G_8$ for the situation with no missing phenotypes, are in Additional file 1: Tables S5, S6.

The genetic gains were higher for traits with a moderate heritability than a low heritability and higher when the residual correlation was negative compared to when it was positive. The SE were high for the whole set of parameters considered, ranging from 0.33 to 0.60, higher for positive residual correlations (average 0.43) and lower for negative residual correlations (average 0.35).

**Fig. 7:**
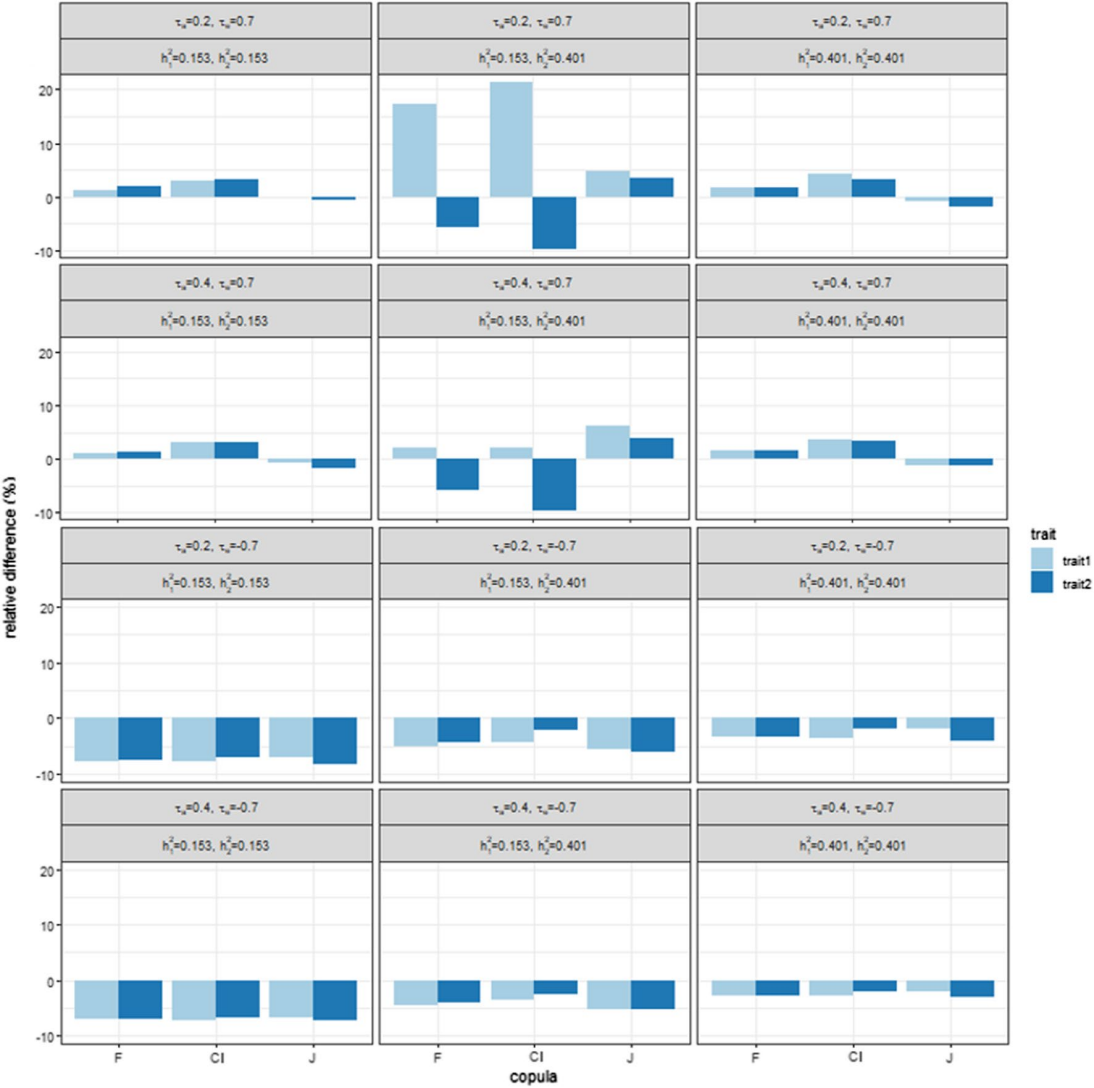
Plot of the relative differences between the normal and non-normal cases in genetic gain for generation $G_8$ with no missing phenotypes. 1000 simulations were performed. Residual copulas are Frank (F), Clayton (Cl) and Joe (J) or the rotated version. Kendall’s correlations $\tau _e$ were $\pm 0.7$. The top two rows represent positive residual correlations and the bottom two rows represent negative residual correlations

Figure 7 shows the relative differences in genetic gain from the normal case for the F, Cl and J copula. When the residual correlation was positive, and the two traits had the same heritability, the genetic gain for F and Cl was higher than for the N copula and lower for the J copula and the relative difference between the Gaussian and non-Gaussian cases was low ($<5\%$). When the heritability of the two traits was different, the genetic gain for the F and Cl copulas was lower for the most heritable trait and higher for the other trait than for the N copula. The genetic gain for the J copula was higher for both traits. The largest absolute relative difference was reached by the Cl copula with a low genetic correlation and high positive residual dependence, with a relative gain (with respect to the Gaussian case) of 21.3% for the trait with a low heritability, and a relative loss of $9.7\%$ for the trait with a moderate heritability. When the residual correlation was negative, the genetic gain was lower for non-normal copulas than for the N copula when the residual correlation and the relative difference with the Gaussian case were moderate (less than 8% absolute relative difference in all cases).

### Summary of the situation with missing phenotypes

Biases and SE of heritabilities and estimated genetic and residual correlations are in Additional file 2: Tables S7, S8.

For the sake of simplicity, we do not present the case of $\tau _e=0.4$ for which biases are similar to the case of $\tau _e=0.7$. For both the estimated heritability and the estimated correlations, biases are extremely low and close to the Gaussian case (always lower than 0.005). The SE ranged from 0.010 to 0.019 for the heritabilities, from 0.023 to 0.049 for the genetic correlation, and from 0.003 to 0.012 for the residual correlations. No significant differences were found from the true parameters in the t-test at level $\alpha =5\%$. Genetic gains are in Additional file 2: Table S9.

As above, the genetic gains for the non-normal copulas were similar to the genetic gains for the N copulas. The maximum absolute difference relative to the Gaussian case corresponded to a loss of 2.2%.

## Discussion

We performed simulation analyses with the bivariate animal model to qualify the robustness of the REML in the presence of deviations from normality due to misspecification of the dependence structure of the residual part. Thus, we looked for classes of non-normal parametric copulas for the residual part that deviate from normality because of the asymmetry and/or because of the tails of the distribution. Hence, we considered:

- A symmetric dependence structure (F copula) and asymmetric distribution (Cl and J copula).
- A case with lower (left) tail dependence (Cl copula) upper (right) tail dependence (J copula) and without tail dependence (F copula).

Note that in the sense of Mardia’s kurtosis, the higher the residual correlation, the heavier the tail of the distribution.

Villanueva et al. [23] reported that the benefit of multivariate BLUP on univariate BLUP is higher when absolute correlation values between traits are high, when genetic and phenotypic correlations have opposite signs, or when heritabilities differ for the two traits. Thus, the parameter sets used in our simulations covered these situations.

We have shown, in the context of a simplified pig breeding scheme, the following three points:

1. With random selection of the reproducers, the REML is robust to a strongly asymmetric and heavy-tailed distribution on the residual part.
2. When the phenotype for one trait is missing for half the population, and selection of the reproducers is carried out among the animals with only one trait by truncation from a combination of their EBV, the REML is also robust to a strongly asymmetric and heavy-tailed distribution on the residual part.
3. With no missing phenotypes and with truncation selection of the reproducers from a combination of their EBV, both the asymmetry of the residual part and the heaviness of the tails of the distribution can lead to significant differences between the theoretical parameters and values obtained by REML, when the correlation of the residual part is sufficiently large. When both the genetic and residual correlations are positive, the differences between the estimated and theoretical heritabilities are larger in the case of unbalanced heritabilities and largest for the trait with the highest heritability. Conversely, the differences between the estimated and theoretical correlations are larger in the case of balanced heritabilities.

As already mentioned above, for the generations with random selection ($G_0$–$G_3$), the robustness of the estimations was remarkable for all the variance-covariance components, in spite of the use of the REML method that uses the likelihood of the multivariate Gaussian distribution to estimate parameters; regardless of whether the residual distribution was asymmetric or heavy-tailed, the estimations were not affected. This can be partially explained by the fact that the probability of the residual part of a chosen animal being close to the center of the bivariate distribution is high in the different models due to the normality of the traits. The centers of non-Gaussian distributions are not really distorted compared to the Gaussian distribution. Hence, neither the asymmetry of the tails nor the heaviness of the tails of the residual distribution affects the REML.

With missing phenotypes on the second trait, we observed no significant impact of a copula miss-specification on REML estimations of the variance-covariance components, even when truncation selection was performed. Since selection was carried out on animals with only one observed trait, the REML was probably comparable to the univariate case, for which the robustness of the REML estimations that face deviation from normality has been reported by many authors, e.g. [24–27].

These two situations (random selection or missing phenotypes) suggest that the observed deviations from normality in the case of truncation selection and two observed traits are due both to the selection process carried out in the upper right tail of the distribution (combined with the asymmetry and the heaviness of the tails of the residual distribution) and indirectly to the selection intensity (see for example [28, 29]), and to the choice of the candidates for breeding. The lowest observed biases for the symmetric non-Gaussian distribution (F copula) suggest that the asymmetry of the residual distribution affects the REML more than the heaviness of tails of the residual distribution.

When the heritability of the two traits is the same, the truncation selection process combined with high (respectively low) dependences in the upper right tail of the residual distribution will lead to overestimation (resp. underestimation) of the residual correlations and consequently to underestimation (respectively overestimation) of the genetic correlations. In the case of a negative correlation, the considered residual distributions do not have a right upper tail, hence the impact on the estimated genetic and residual correlations was globally lower (in particular, SE of the genetic correlation were greater). Nevertheless, in this case, the selection had more impact on the REML estimations of the heritabilities (with smaller SE), particularly when the heritability of the two traits was moderate.

The largest differences in genetic gain compared with the Gaussian case were for the Cl copula with unbalanced heritabilities, for which the variability in the upper left part of the distribution was the highest among the copulas considered here.

Copulas do not enable characterization of the full dependence structure between random vectors, except for a few copulas (e.g. normal or t-copulas). See Hofert et al. [30] for an exhaustive discussion on this topic. Hence, to simulate non-Gaussian phenotypes, non-Gaussian dependence structures were only applied to the residuals.

We limited our study to two correlated traits. The case of $d>2$ dimensional phenotypes could be explored with a more complex dependence structure between phenotypes. For example, one could study hierarchical Archimedean copulas in multivariate residuals by considering the composition of bivariate Archimedean copulas, see for example, Okhrin and Ristig [31].

In this study, our aim was to exacerbate differences between the Gaussian and non-Gaussian case by considering high residual (and phenotypic) correlations and heavy-tailed distributions for the residual part. Of course, in practice, differences between the true distribution and the Gaussian distribution are not always so pronounced and any errors in the estimation of the genetic components can be difficult to evaluate. For bivariate data, the scatter plot of the ranks (based on the phenotypes, corrected for fixed effects) can allow to identify the copula, and in particular, it can be viewed as a graphical tool to check the normality of the copula, especially the asymmetry or the tail dependence of the data. Mardia’s skewness and kurtosis allow to indicate deviations from the normality and can be evaluated before the selection process to anticipate estimation bias. Other measures can be used to evaluate the deviation from the normality. For example, lower/upper tail dependencies can be evaluated below/above a cut-off parameter according to Schmid and Schmidt [32].

To reduce biases due to the selection process and the miss-copula specification in the REML estimations, an inference copula-based method, which assumes that the copula family of the residual part is known, could be developed to estimate more accurately the parameters of the genetic model. Nevertheless, this assumes prior knowledge on the residual dependence structure (for example biological evidence); the choice for the parametric copula on the residual part could also be guided by the scatter plot of the ranks. An interesting perspective would be to propose an efficient algorithm to evaluate the parameters of the mixed model when the copula of the residual part is not normal.

## Conclusions

In this paper, we used simulations to assess the impact of a non-Gaussian distribution of the residuals for the bivariate animal model on the REML estimation of the variance-covariance terms. We considered one situation in which two traits are observed for each animal and another situation in which the second trait is only observed in half of the population (typically measured after culling). In the two situations, when male and female breeders are chosen at random, our results show that even in the case of a non-Gaussian distribution for the residual part, REML estimations based on Gaussian assumptions provide robust estimates of variance-covariance components. For a truncation selection based on the summation of the EBV, in the case of missing phenotypes for the second trait, we observed no impact of the miss-specification on the REML estimations of the variance-covariance components. Thus, in this situation, REML can be used even when phenotypes deviate from the multi-normal distribution. Nevertheless, in the case of two observed phenotypes, one must pay attention to possible errors due to the residual dependence structures, particularly in the case of high correlations and asymmetric or heavy-tailed distributions.

## Supplementary Information

**Additional file 1: Table S1**. Bias and SE of estimated heritabilities, for positive residual dependence,with no missing phenotypes.** Table S2**. Bias and SE of estimated heritabilities, for negative residual dependence,with no missing phenotypes.** Table S3**. Bias and SE of estimated genetic and residual correlations, for positive residual dependence, with no missing phenotypes. **Table S4**. Bias and SE of estimated genetic and residual correlations, for negative residual dependence, with no missing phenotypes. **Table S5**. Mean value and SE of the genetic gains for G8, for positive residual dependence, with no missing phenotypes. **Table S6**. Mean value and SE of the genetic gains for G8, for negative residual dependence, with no missing phenotypes.

**Additional file 2: Table S7**. Bias and SE of estimated heritabilities, with missing phenotypes. **Table S8**. Bias and SE of estimated genetic and residual correlations, with missing phenotypes. **Table S9**. Mean value and SE of the genetic gain for G_8_, with missing phenotypes.

## Data Availability

The datasets generated during the current study are available from the corresponding author on reasonable request.
