# Supplementary material for: Copula miss-specification in REML multivariate genetic animal model estimation
Source: Genet Sel Evol. 2022 May 26;54:36. doi: 10.1186/s12711-022-00729-3 (PMC9137146; doi:10.1186/s12711-022-00729-3)
Supplement: Supplementary file 1 — Additional file 1: Table S1. Bias and SE of estimated heritabilities, for positive residual dependence,with no missing phenotypes. Table S2. Bias and SE of estimated heritabilities, for negative residual dependence,with no missing phenotypes. Table S3. Bias and SE of estimated genetic and residual correlations, for positive residual dependence, with no missing phenotypes. Table S4. Bias and SE of estimated genetic and residual correlations, for negative residual dependence, with no missing phenotypes. Table S5. Mean value and SE of the genetic gains for G8, for positive residual dependence, with no missing phenotypes. Table S6. Mean value and SE of the genetic gains for G8, for negative residual dependence, with no missing phenotypes. [file 12711_2022_729_MOESM1_ESM.pdf]

Copula miss-specification in REML multivariate genetic animal model estimation
Additional file 1

Tom Rohmer*[,1], Anne Ricard[2,3], and Ingrid David[1]

[1]GenPhySE, Université de Toulouse, INRAE, ENVT, F-31326, Castanet Tolosa, France
[2]Université Paris-Saclay, INRAE, AgroParisTech, GABI, Jouy-en-Josas, France
[3]Institut Français du Cheval et de l'Equitation, Pôle Développement, Innovation et Recherche, Exmes, France

Table S1: Bias and SE of estimated heritabilities, for positive residual dependence, with no missing phenotypes.

True parameters					Estimated heritabilities							
					Trait 1				Trait 2			
h_1^2	h_2^2	τ_a	τ_e		N	F	Cl	J	N	F	Cl	J
0.153	0.153	0.2	0.4	bias	0.002	0.003	0.004	0.004	0.002	0.004	0.004	0.003
				SE	0.013	0.013	0.012	0.014	0.013	0.013	0.013	0.014
0.153	0.401	0.2	0.4	bias	0.001	0.004	0.010	-0.010	0.004	-0.003	-0.015	0.036
				SE	0.013	0.014	0.014	0.013	0.016	0.016	0.015	0.017
0.401	0.401	0.2	0.4	bias	0.004	0.008	0.010	0.014	0.003	0.008	0.010	0.013
				SE	0.016	0.017	0.016	0.019	0.017	0.017	0.017	0.019
0.153	0.153	0.4	0.4	bias	0.003	0.004	0.008	-0.002	0.002	0.005	0.009	-0.002
				SE	0.012	0.013	0.012	0.013	0.012	0.013	0.013	0.012
0.153	0.401	0.4	0.4	bias	0.001	0.005	0.013	-0.010	0.004	-0.001	-0.009	0.029
				SE	0.013	0.014	0.014	0.012	0.016	0.017	0.015	0.017
0.401	0.401	0.4	0.4	bias	0.003	0.011	0.020	-0.003	0.003	0.012	0.021	-0.003
				SE	0.016	0.017	0.016	0.018	0.017	0.017	0.017	0.018
0.153	0.153	0.2	0.7	bias	0.002	-0.001	-0.004	0.018	0.002	0.000	-0.004	0.019
				SE	0.012	0.012	0.011	0.017	0.012	0.012	0.011	0.016
0.153	0.401	0.2	0.7	bias	0.001	0.005	0.015	-0.015	0.004	-0.023	-0.052*	0.057*
				SE	0.014	0.015	0.015	0.013	0.013	0.015	0.014	0.015
0.401	0.401	0.2	0.7	bias	0.004	-0.000	-0.004	0.027	0.004	0.000	-0.004	0.027
				SE	0.016	0.016	0.016	0.017	0.015	0.016	0.016	0.018
0.153	0.153	0.4	0.7	bias	0.002	0.002	0.002	0.010	0.002	0.003	0.003	0.009
				SE	0.012	0.012	0.011	0.015	0.012	0.012	0.012	0.014
0.153	0.401	0.4	0.7	bias	0.001	0.004	0.012	-0.006	0.004	-0.024	-0.051*	0.059*
				SE	0.014	0.015	0.015	0.012	0.014	0.015	0.014	0.015
0.401	0.401	0.4	0.7	bias	0.004	0.006	0.008	0.020	0.003	0.005	0.009	0.020
				SE	0.016	0.016	0.016	0.017	0.016	0.016	0.016	0.018

Biases and SEs were obtained from 1 000 simulations, using G_1 to G_8. Residual copulas were normal(N), Frank(F), Clayton(Cl) and Joe(J). '*': significant difference between estimated and true heritability for the t-test at level $\alpha = 0.05$

*Corresponding author: tom.rohmer@inrae.fr

Table S2: Bias and SE of estimated heritabilities, for negative residual dependence, with no missing phenotypes.

True parameters					Estimated heritability							
					Trait 1				Trait 2			
h_1^2	h_2^2	τ_a	τ_e		N	F	Cl	J	N	F	Cl	J
0.153	0.153	0.2	-0.4	bias	0.002	-0.003	-0.013	0.012	0.002	-0.003	0.011	-0.014
				SE	0.011	0.011	0.010	0.013	0.011	0.011	0.013	0.010
0.153	0.401	0.2	-0.4	bias	0.002	-0.003	-0.012	0.010	0.004	-0.010	0.035*	-0.035*
				SE	0.012	0.012	0.011	0.014	0.014	0.015	0.016	0.014
0.401	0.401	0.2	-0.4	bias	0.003	-0.008	-0.033*	0.036*	0.004	-0.008	0.035*	-0.036*
				SE	0.016	0.016	0.015	0.018	0.015	0.016	0.017	0.015
0.153	0.153	0.4	-0.4	bias	0.002	-0.004	-0.012	0.007	0.002	-0.004	0.007	-0.013
				SE	0.010	0.010	0.009	0.012	0.010	0.010	0.011	0.009
0.153	0.401	0.4	-0.4	bias	0.002	-0.003	-0.010	0.005	0.004	-0.010	0.028	-0.034*
				SE	0.011	0.011	0.009	0.012	0.013	0.014	0.015	0.013
0.401	0.401	0.4	-0.4	bias	0.004	-0.007	-0.028*	0.027	0.004	-0.007	0.026	-0.030*
				SE	0.014	0.015	0.014	0.016	0.013	0.014	0.015	0.013
0.153	0.153	0.2	-0.7	bias	0.002	-0.009	-0.026*	0.019	0.002	-0.009	0.019	-0.026*
				SE	0.010	0.010	0.009	0.014	0.010	0.010	0.013	0.009
0.153	0.401	0.2	-0.7	bias	0.002	-0.005	-0.018	0.022	0.004	-0.016	0.043*	-0.055*
				SE	0.012	0.012	0.010	0.015	0.011	0.013	0.016	0.012
0.401	0.401	0.2	-0.7	bias	0.004	-0.009	-0.047*	0.049*	0.004	-0.009	0.049*	-0.048*
				SE	0.014	0.015	0.014	0.017	0.014	0.015	0.016	0.013
0.153	0.153	0.4	-0.7	bias	0.002	-0.009	-0.024*	0.010	0.002	-0.009	0.011	-0.025*
				SE	0.009	0.009	0.008	0.012	0.008	0.009	0.011	0.008
0.153	0.401	0.4	-0.7	bias	0.002	-0.004	-0.017*	0.016	0.005	-0.013	0.032*	-0.047*
				SE	0.010	0.010	0.009	0.013	0.010	0.012	0.014	0.011
0.401	0.401	0.4	-0.7	bias	0.005	-0.006	-0.038*	0.038*	0.004	-0.006	0.037*	-0.039*
				SE	0.013	0.013	0.012	0.015	0.012	0.013	0.014	0.011

Biases and SEs were obtained from 1 000 simulations, using G_1 to G_8. Residual copulas were normal(N), Frank(F), Clayton(Cl) and Joe(J). '*': significant difference between estimated and true heritability for the t-test at level $\alpha = 0.05$

Table S3: Bias and SE of estimated genetic and residual correlations, for positive residual dependence, with no missing phenotypes.

True parameters					Estimated parameters							
					genetic correlations				residual correlations			
h_1^2	h_2^2	τ_a	τ_e		N	F	Cl	J	N	F	Cl	J
0.153	0.153	0.2	0.4	bias	-0.003	0.020	0.073	-0.093	-0.000	-0.002	-0.009	0.011
				SE	0.055	0.055	0.052	0.057	0.007	0.007	0.007	0.008
0.153	0.401	0.2	0.4	bias	0.000	0.020	0.062	-0.051	-0.001	-0.006	-0.019*	0.019*
				SE	0.042	0.041	0.039	0.042	0.009	0.009	0.009	0.009
0.401	0.401	0.2	0.4	bias	-0.000	0.031	0.100*	-0.141*	-0.001	-0.012	-0.038*	0.057*
				SE	0.033	0.032	0.031	0.037	0.011	0.012	0.012	0.015
0.153	0.153	0.4	0.4	bias	-0.001	0.015	0.052	-0.060	-0.000	-0.002	-0.006	0.007
				SE	0.043	0.042	0.038	0.049	0.006	0.007	0.007	0.007
0.153	0.401	0.4	0.4	bias	0.001	0.013	0.039	-0.033	-0.000	-0.004	-0.013	0.015
				SE	0.032	0.032	0.029	0.035	0.008	0.008	0.008	0.008
0.401	0.401	0.4	0.4	bias	-0.000	0.021	0.068*	-0.101*	-0.000	-0.009	-0.029*	0.038*
				SE	0.025	0.024	0.022	0.031	0.010	0.011	0.011	0.012
0.153	0.153	0.2	0.7	bias	-0.005	0.048	0.130*	-0.161*	-0.001	-0.006	-0.016*	0.023*
				SE	0.051	0.051	0.046	0.058	0.003	0.004	0.004	0.007
0.153	0.401	0.2	0.7	bias	-0.003	0.009	0.059	0.031	-0.001	-0.012*	-0.031*	0.013*
				SE	0.038	0.038	0.038	0.036	0.004	0.005	0.006	0.006
0.401	0.401	0.2	0.7	bias	-0.003	0.060*	0.140*	-0.128*	-0.001	-0.025*	-0.056*	0.060*
				SE	0.029	0.030	0.027	0.032	0.006	0.008	0.007	0.009
0.153	0.153	0.4	0.7	bias	-0.003	0.034	0.089*	-0.108*	-0.000	-0.004	-0.010*	0.013*
				SE	0.035	0.034	0.031	0.044	0.003	0.003	0.003	0.005
0.153	0.401	0.4	0.7	bias	-0.001	0.003	0.030	0.039	-0.000	-0.009*	-0.023*	0.011*
				SE	0.027	0.028	0.026	0.026	0.004	0.005	0.005	0.005
0.401	0.401	0.4	0.7	bias	-0.001	0.049*	0.106*	-0.128*	-0.001	-0.018*	-0.040*	0.052*
				SE	0.021	0.020	0.018	0.027	0.005	0.006	0.006	0.008

Biases and SEs were obtained from 1 000 simulations, using G_1 to G_8. Residual copulas were normal(N), Frank(F), Clayton(Cl) and Joe(J). True genetic correlations are $\rho_a \in \{0.309, 0.588\}$. True residual correlations for N, F, Cl and J for $\tau_e = 0.4$ respectively are 0.588, 0.544, 0.578 and 0.576 and for $\tau_e = 0.7$ respectively are 0.891, 0.846, 0.852 and 0.850. '*': significant difference between estimated and true correlation for the t-test at level $\alpha = 0.05$

Table S4: Bias and SE of estimated genetic and residual correlations, for negative residual dependence, with no missing phenotypes.

True parameters					Estimated parameters							
					genetic correlations				residual correlations			
h_1^2	h_2^2	τ_a	τ_e		N	F	Cl	J	N	F	Cl	J
0.153	0.153	0.2	-0.4	bias	0.005	-0.011	-0.027	-0.034	0.001	0.006	0.006	0.007
				SE	0.068	0.069	0.070	0.072	0.007	0.008	0.008	0.008
0.153	0.401	0.2	-0.4	bias	0.005	-0.009	-0.028	-0.031	0.002	0.011	0.003	0.020*
				SE	0.053	0.051	0.052	0.052	0.010	0.010	0.010	0.010
0.401	0.401	0.2	-0.4	bias	0.002	-0.005	-0.025	-0.029	0.002	0.015	0.015	0.018
				SE	0.039	0.040	0.039	0.041	0.013	0.013	0.013	0.013
0.153	0.153	0.4	-0.4	bias	0.006	-0.007	-0.018	-0.023	0.002	0.007	0.008	0.009
				SE	0.060	0.061	0.063	0.063	0.008	0.008	0.008	0.008
0.153	0.401	0.4	-0.4	bias	0.004	-0.007	-0.016	-0.024	0.002	0.013	0.004	0.023*
				SE	0.047	0.049	0.047	0.049	0.010	0.010	0.011	0.010
0.401	0.401	0.4	-0.4	bias	0.003	-0.004	-0.016	-0.020	0.002	0.018	0.016	0.019
				SE	0.035	0.035	0.035	0.034	0.013	0.014	0.014	0.014
0.153	0.153	0.2	-0.7	bias	0.005	-0.018	-0.093	-0.096	0.001	0.012*	0.016*	0.016*
				SE	0.071	0.071	0.074	0.075	0.004	0.005	0.005	0.005
0.153	0.401	0.2	-0.7	bias	0.008	-0.002	-0.100	-0.022	0.001	0.016*	0.014	0.026*
				SE	0.054	0.054	0.057	0.052	0.005	0.007	0.007	0.007
0.401	0.401	0.2	-0.7	bias	0.006	0.006	-0.031	-0.035	0.001	0.016	0.018	0.019*
				SE	0.039	0.040	0.041	0.041	0.006	0.009	0.009	0.010
0.153	0.153	0.4	-0.7	bias	0.008	-0.010	-0.070	-0.071	0.001	0.014*	0.018*	0.019*
				SE	0.064	0.064	0.071	0.073	0.004	0.005	0.005	0.005
0.153	0.401	0.4	-0.7	bias	0.007	-0.002	-0.062	-0.026	0.001	0.017*	0.015	0.028*
				SE	0.049	0.049	0.054	0.048	0.005	0.008	0.008	0.008
0.401	0.401	0.4	-0.7	bias	0.004	0.005	-0.020	-0.023	0.001	0.016	0.018	0.019
				SE	0.036	0.035	0.036	0.037	0.007	0.010	0.010	0.010

Biases and SEs were obtained from 1 000 simulations, using G_1 to G_8. Residual copulas were normal(N), Frank(F), Clayton(Cl) and Joe(J) or rotated version. True genetic correlations are $\rho_a \in \{0.309, 0.588\}$. True residual correlations for N, F, Cl and J (or rotated version), for $\tau_e = -0.4$ respectively are -0.588, -0.544, -0.578 and -0.576 and for $\tau_e = -0.7$ respectively are -0.891, -0.846, -0.852 and -0.850. '*': significant difference between estimated and true correlation for the t-test at level $\alpha = 0.05$

Table S5: Mean value and SE of the genetic gains for G_8, for positive residual dependence, with no missing phenotypes.

True parameters					genetic gain							
					trait 1				trait 2			
h_1^2	h_2^2	τ_a	τ_e		N	F	Cl	J	N	F	Cl	J
0.153	0.153	0.2	0.4	mean	2.781	2.819	2.890	2.708	2.808	2.845	2.906	2.693
				SE	0.491	0.475	0.493	0.475	0.481	0.490	0.475	0.474
0.153	0.401	0.2	0.4	mean	1.878	1.962	1.972	1.858	4.788	4.722	4.714	4.905
				SE	0.460	0.454	0.447	0.456	0.367	0.372	0.352	0.356
0.401	0.401	0.2	0.4	mean	3.761	3.826	3.937	3.608	3.761	3.831	3.923	3.612
				SE	0.419	0.415	0.401	0.423	0.415	0.410	0.404	0.425
0.153	0.153	0.4	0.4	mean	3.211	3.248	3.335	3.107	3.231	3.271	3.350	3.099
				SE	0.444	0.440	0.444	0.441	0.427	0.446	0.428	0.418
0.153	0.401	0.4	0.4	mean	3.096	3.116	3.131	3.100	4.824	4.753	4.763	4.884
				SE	0.431	0.418	0.428	0.414	0.368	0.366	0.352	0.351
0.401	0.401	0.4	0.4	mean	4.310	4.376	4.510	4.143	4.316	4.383	4.501	4.137
				SE	0.384	0.378	0.374	0.392	0.380	0.378	0.365	0.396
0.153	0.153	0.2	0.7	mean	2.655	2.690	2.733	2.655	2.677	2.733	2.766	2.660
				SE	0.562	0.508	0.521	0.582	0.546	0.545	0.503	0.555
0.153	0.401	0.2	0.7	mean	1.186	1.390	1.439	1.243	5.483	5.166	4.949	5.673
				SE	0.473	0.477	0.474	0.464	0.346	0.362	0.355	0.336
0.401	0.401	0.2	0.7	mean	3.611	3.674	3.762	3.581	3.631	3.689	3.747	3.565
				SE	0.450	0.441	0.418	0.450	0.439	0.426	0.421	0.448
0.153	0.153	0.4	0.7	mean	3.081	3.117	3.174	3.059	3.103	3.142	3.200	3.048
				SE	0.477	0.468	0.455	0.469	0.468	0.475	0.441	0.463
0.153	0.401	0.4	0.7	mean	2.659	2.715	2.712	2.823	5.426	5.112	4.907	5.636
				SE	0.445	0.443	0.441	0.410	0.340	0.359	0.354	0.333
0.401	0.401	0.4	0.7	mean	4.162	4.230	4.309	4.107	4.162	4.231	4.306	4.107
				SE	0.401	0.403	0.389	0.400	0.404	0.398	0.378	0.407

Means and SEs were obtained from 1 000 simulations. Residual copulas were normal(N), Frank(F), Clayton(Cl) and Joe(J).

Table S6: Mean value and SE of the genetic gains for G_8, for negative residual dependence, with no missing phenotypes.

True parameters					genetic gain							
					trait 1				trait 2			
h_1^2	h_2^2	τ_a	τ_e		N	F	Cl	J	N	F	Cl	J
0.153	0.153	0.2	-0.4	mean	3.783	3.637	3.692	3.709	3.797	3.651	3.731	3.679
				SE	0.386	0.394	0.387	0.406	0.386	0.401	0.396	0.390
0.153	0.401	0.2	-0.4	mean	3.401	3.286	3.417	3.299	5.195	5.048	5.286	4.980
				SE	0.388	0.409	0.391	0.391	0.339	0.353	0.339	0.356
0.401	0.401	0.2	-0.4	mean	4.796	4.671	4.707	4.791	4.810	4.692	4.789	4.697
				SE	0.354	0.366	0.367	0.366	0.353	0.360	0.360	0.348
0.153	0.153	0.4	-0.4	mean	4.333	4.166	4.240	4.243	4.345	4.195	4.252	4.227
				SE	0.373	0.375	0.374	0.390	0.364	0.371	0.372	0.376
0.153	0.401	0.4	-0.4	mean	4.462	4.333	4.479	4.311	5.466	5.312	5.516	5.252
				SE	0.365	0.375	0.356	0.365	0.330	0.342	0.330	0.340
0.401	0.401	0.4	-0.4	mean	5.433	5.307	5.366	5.388	5.449	5.320	5.392	5.342
				SE	0.341	0.344	0.344	0.347	0.332	0.336	0.344	0.336
0.153	0.153	0.2	-0.7	mean	4.814	4.441	4.437	4.470	4.829	4.459	4.485	4.426
				SE	0.356	0.369	0.375	0.380	0.351	0.356	0.367	0.361
0.153	0.401	0.2	-0.7	mean	4.233	4.019	4.051	3.991	6.074	5.801	5.939	5.709
				SE	0.360	0.374	0.362	0.375	0.313	0.332	0.326	0.329
0.401	0.401	0.2	-0.7	mean	5.509	5.328	5.303	5.402	5.518	5.336	5.409	5.292
				SE	0.333	0.339	0.342	0.344	0.326	0.337	0.329	0.330
0.153	0.153	0.4	-0.7	mean	5.443	5.069	5.050	5.074	5.456	5.075	5.088	5.055
				SE	0.336	0.347	0.352	0.359	0.337	0.338	0.351	0.344
0.153	0.401	0.4	-0.7	mean	5.342	5.095	5.149	5.060	6.357	6.098	6.204	6.020
				SE	0.335	0.346	0.345	0.351	0.307	0.316	0.317	0.318
0.401	0.401	0.4	-0.7	mean	6.140	5.969	5.963	6.010	6.143	5.979	6.015	5.949
				SE	0.314	0.316	0.317	0.318	0.303	0.324	0.323	0.314

Means and SEs were obtained from 1 000 simulations. Residual copulas were normal(N), Frank(F), Clayton(Cl) and Joe(J).
