# Supplementary material for: Copula miss-specification in REML multivariate genetic animal model estimation
Source: Genet Sel Evol. 2022 May 26;54:36. doi: 10.1186/s12711-022-00729-3 (PMC9137146; doi:10.1186/s12711-022-00729-3)
Supplement: Supplementary file 2 — Additional file 2: Table S7. Bias and SE of estimated heritabilities, with missing phenotypes. Table S8. Bias and SE of estimated genetic and residual correlations, with missing phenotypes. Table S9. Mean value and SE of the genetic gain for G8, with missing phenotypes. [file 12711_2022_729_MOESM2_ESM.pdf]

Copula miss-specification in REML multivariate genetic animal model estimation
Additional file 2

Tom Rohmer*,[1], Anne Ricard[2,3], and Ingrid David[1]

[1]GenPhySE, Université de Toulouse, INRAE, ENVT, F-31326, Castanet Tolosa, France
[2]Université Paris-Saclay, INRAE, AgroParisTech, GABI, Jouy-en-Josas, France
[3]Institut Français du Cheval et de l'Equitation, Pôle Développement, Innovation et Recherche, Exmes, France

Table S7: Bias and SE of estimated heritabilities, with missing phenotypes.

True parameters					Estimated heritabilities							
					Trait 1				Trait 2			
h_1^2	h_2^2	τ_a	τ_e		N	F	Cl	J	N	F	Cl	J
0.153	0.153	0.2	0.7	bias	0.002	0.002	0.003	0.003	0.002	0.002	0.002	0.002
				SE	0.010	0.010	0.010	0.010	0.012	0.013	0.012	0.013
0.153	0.401	0.2	0.7	bias	0.002	0.002	0.003	0.003	-0.002	0.000	0.000	-0.000
				SE	0.011	0.011	0.010	0.011	0.018	0.018	0.018	0.018
0.401	0.153	0.2	0.7	bias	0.002	0.001	0.003	0.002	0.002	0.002	0.002	0.002
				SE	0.014	0.014	0.014	0.014	0.013	0.013	0.012	0.013
0.401	0.401	0.2	0.7	bias	0.002	0.002	0.003	0.003	0.001	0.002	0.001	0.002
				SE	0.015	0.015	0.014	0.015	0.019	0.019	0.019	0.019
0.153	0.153	0.4	0.7	bias	0.002	0.003	0.003	0.003	0.002	0.003	0.002	0.002
				SE	0.010	0.010	0.010	0.010	0.012	0.012	0.012	0.012
0.153	0.401	0.4	0.7	bias	0.002	0.002	0.002	0.002	0.001	0.001	0.001	0.001
				SE	0.010	0.010	0.010	0.010	0.017	0.019	0.018	0.018
0.401	0.153	0.4	0.7	bias	0.002	0.002	0.002	0.003	0.002	0.002	0.002	0.002
				SE	0.015	0.015	0.014	0.014	0.012	0.012	0.011	0.012
0.401	0.401	0.4	0.7	bias	0.003	0.002	0.003	0.003	0.002	0.002	0.002	0.003
				SE	0.015	0.015	0.015	0.014	0.017	0.018	0.017	0.018

Biases and SEs were obtained from 1 000 simulations, using G_1 to G_8. Residual copulas were normal(N), Frank(F), Clayton(Cl) and Joe(J)

*Corresponding author: tom.rohmer@inrae.fr

Table S8: Bias and SE of estimated genetic and residual correlations, with missing phenotypes.

True parameters					Estimated parameters							
					genetic correlations				residual correlations			
h_1^2	h_2^2	τ_a	τ_e		N	F	Cl	J	N	F	Cl	J
0.153	0.153	0.2	0.7	bias	0.000	0.001	-0.001	-0.001	-0.001	-0.001	-0.001	-0.000
				SE	0.046	0.049	0.047	0.048	0.004	0.005	0.005	0.005
0.153	0.401	0.2	0.7	bias	0.002	0.003	0.003	0.002	-0.005	-0.004	-0.004	-0.004
				SE	0.040	0.041	0.040	0.040	0.008	0.008	0.008	0.008
0.401	0.153	0.2	0.7	bias	0.004	0.001	0.002	0.001	-0.003	-0.002	-0.002	-0.002
				SE	0.038	0.039	0.038	0.038	0.007	0.008	0.008	0.008
0.401	0.401	0.2	0.7	bias	0.004	0.004	0.004	0.005	-0.005	-0.005	-0.005	-0.004
				SE	0.032	0.033	0.032	0.033	0.010	0.011	0.012	0.011
0.153	0.153	0.4	0.7	bias	-0.000	0.001	-0.001	-0.002	-0.001	-0.000	-0.000	0.000
				SE	0.033	0.036	0.035	0.034	0.003	0.004	0.004	0.004
0.153	0.401	0.4	0.7	bias	0.003	0.001	0.000	0.000	-0.003	-0.002	-0.002	-0.002
				SE	0.029	0.030	0.029	0.029	0.006	0.006	0.006	0.006
0.401	0.153	0.4	0.7	bias	0.002	0.001	0.002	0.000	-0.002	-0.001	-0.001	-0.001
				SE	0.030	0.030	0.029	0.028	0.006	0.006	0.006	0.006
0.401	0.401	0.4	0.7	bias	0.003	0.002	0.002	0.001	-0.003	-0.002	-0.002	-0.001
				SE	0.023	0.025	0.024	0.024	0.007	0.008	0.008	0.008

Biases and SEs were obtained from 1 000 simulations, using G_1 to G_8. Residual copulas were normal(N), Frank(F), Clayton(Cl) and Joe(J). True genetic correlations are $\rho_a \in \{0.309, 0.588\}$. True residual correlations for N, F, Cl and J for $\tau_e = 0.4$ respectively are 0.588, 0.544, 0.578 and 0.576 and for $\tau_e = 0.7$ respectively are 0.891, 0.846, 0.852 and 0.850.

Table S9: Mean value and SE of the genetic gain for G_8, with missing phenotypes.

True parameters					genetic gain							
					Trait 1				Trait 2			
h_1^2	h_2^2	τ_a	τ_e		N	F	Cl	J	N	F	Cl	J
0.153	0.153	0.2	0.7	mean	3.194	3.217	3.224	3.229	2.342	2.333	2.311	2.327
				SE	0.439	0.455	0.441	0.457	0.463	0.472	0.466	0.466
0.153	0.401	0.2	0.7	mean	2.525	2.580	2.573	2.561	3.172	3.143	3.146	3.146
				SE	0.486	0.461	0.445	0.465	0.415	0.444	0.417	0.422
0.401	0.153	0.2	0.7	mean	4.765	4.755	4.772	4.757	2.074	2.069	2.074	2.060
				SE	0.370	0.364	0.364	0.371	0.421	0.431	0.433	0.425
0.401	0.401	0.2	0.7	mean	4.346	4.347	4.353	4.362	2.806	2.795	2.797	2.798
				SE	0.391	0.384	0.381	0.395	0.413	0.420	0.413	0.421
0.153	0.153	0.4	0.7	mean	3.470	3.482	3.479	3.488	2.920	2.911	2.888	2.914
				SE	0.418	0.425	0.410	0.436	0.432	0.447	0.436	0.445
0.153	0.401	0.4	0.7	mean	3.166	3.190	3.170	3.184	3.525	3.468	3.466	3.482
				SE	0.422	0.425	0.425	0.437	0.389	0.416	0.409	0.406
0.401	0.153	0.4	0.7	mean	4.840	4.833	4.836	4.833	3.209	3.208	3.203	3.190
				SE	0.376	0.357	0.361	0.364	0.418	0.399	0.410	0.414
0.401	0.401	0.4	0.7	mean	4.610	4.613	4.613	4.605	3.643	3.638	3.637	3.634
				SE	0.376	0.376	0.382	0.382	0.396	0.394	0.395	0.390

Means and SEs were obtained from 1 000 simulations. Residual copulas were normal(N), Frank(F), Clayton(Cl) and Joe(J).
